# PPAR Action in Human Placental Development and Pregnancy and Its Complications

**DOI:** 10.1155/2008/527048

**Published:** 2007-12-30

**Authors:** Fritz Wieser, Leslie Waite, Christophe Depoix, Robert N. Taylor

**Affiliations:** Department of Gynecology and Obstetrics, Emory University School of Medicine, Atlanta, GA 30322, USA

## Abstract

During pregnancy crucial anatomic, physiologic, and metabolic changes challenge the mother and the fetus. The placenta is a remarkable organ that allows the mother and the fetus to adapt to the new metabolic, immunologic, and angiogenic environment imposed by gestation. One of the physiologic systems that appears to have evolved to sustain this metabolic regulation is mediated by peroxisome proliferator-activated receptors (PPARs). In clinical pregnancy-specific disorders, including preeclampsia, gestational diabetes, and intrauterine growth restriction, aberrant regulation of components of the PPAR system parallels dysregulation of metabolism, inflammation and angiogenesis. This review summarizes current knowledge on the role of PPARs in regulating human trophoblast invasion, early placental development, and also in the physiology of clinical pregnancy and its complications. As increasingly indicated in the literature, pregnancy disorders, such as preeclampsia and gestational diabetes, represent potential targets for treatment with PPAR ligands. With the advent of more specific PPAR agonists that exhibit efficacy in ameliorating metabolic, inflammatory, and angiogenic disturbances, further studies of their application in pregnancy-related diseases are warranted.

## 1. INTRODUCTION

Peroxisome proliferator-activated receptors (PPARs) are major regulators of lipid and glucose metabolism, inflammation, and angiogenesis [1–6] that allow adaptation of the mother to the nutritional and perfusion requirements of the fetus [3, 7, 8]. PPARs, members of the nuclear hormone receptor superfamily, are ligand-activated transcription factors. The PPAR amino acid sequence can be divided into five modular domains: A/B, C, D, E, and F. Domain E is the ligand binding domain (LBD) and contains a ligand-dependent transcriptional activation function (AF-2). Domain C is the DNA binding domain, formed of two typical zinc fingers. PPARs activate DNA direct repeat response elements by binding as heterodimers with retinoic acid receptor (RXR) partners [9]. There are three PPAR isotypes, PPAR*α*, PPAR*γ*, and PPAR*β*/*δ*, that are highly conserved across species, with mouse, rat, and human sequences sharing >80% amino acid homology [6, 10]. The conserved expression of different PPAR and RXR isotypes in both rat and human placentas [11] suggests that these receptors play functional roles in placental lipid transfer and homeostasis. PPAR*α* has a wide distribution and is prominent in tissues with high metabolic rates such as liver, heart, skeletal muscle, and kidney and in steroidogenic organs such as the adrenals [12]. PPAR*γ* has three isoforms (PPAR*γ*1, *γ*2, and *γ*3) and is expressed in brown and white adipose tissue, large intestine, to a lesser extent in immune cells (monocytes, macrophages, Peyer’s patches of the digestive tract), the mucosa of colon and cecum, and placental trophoblasts [13– 16]. PPAR*β*/*δ* is distributed in all tissues tested with particularly high expression in placenta and large intestine [8, 17, 18]. PPAR*α* and PPAR*γ* are involved in adipocyte differentiation, lipid metabolism, insulin action, and in the regulation of inflammatory responses [1, 5, 16], particularly involving the macrophage [19]. PPAR*β*/*δ* is known to be involved in lipid metabolism and inflammation, as well as keratinocyte differentiation and wound healing [5, 20, 21].

The PPAR system is intimately involved in cardiovascular disease, obesity, as well as pregnancy-specific diseases [6, 22]. Over the past decade studies have shown that all three PPAR isotypes are expressed in human placental trophoblast cells [11] and that they are involved in the regulation of pregnancy physiology and its clinical complications. Physiological and pathophysiological conditions that modulate the PPAR system [22– 35] influence the risk and course of preeclampsia (PE), gestational diabetes mellitus (GDM), or intrauterine growth restriction (IUGR) [36– 53]. Some of these diseases and factors involving the PPAR system are summarized in Tables 1 and 2.

In early pregnancy, immediately after embryonic implantation, major maternal physiologic changes occur in the cardiovascular, hepatic, and endocrine systems with resultant anatomical and metabolic modifications that serve to promote maternal immune tolerance of the conceptus and to provide the fetus with its increased nutritional needs [54, 55]. Metabolic changes (including increased availability of glucose, low density lipoprotein, and fatty acids) increased insulin resistance and altered amino acid metabolism, immunologic, and hematologic changes (including an increase in plasma volume). Establishment of a thrombophilic state and extensive placental and decidual angiogenesis are observed in pregnancy, and these changes require a complex activation of regulating mediators [56–58].

Pregnancy complications result when the mother and/or fetus fail to adapt to these new metabolic, angiogenic, and thrombogenic challenges. Women with preexisting compromise to their vascular homeostasis, such as underlying hypertension, diabetes mellitus, or metabolic syndrome, have a significantly increased risk of developing pregnancy complications (see Table 2). Placenta-associated complications also can lead to impaired growth or fetal demise [59, 60]. These placental conditions share vasculopathological mechanisms in common with atherosclerosis and represent early markers for maternal risk of cardiovascular disease [61, 62] and hypertension [61,63, 64]. Curiously, a prior history of preeclampsia appears to confer protection against the future development of endometriosis and some cancers [65, 66].

PPARs can be activated by natural ligands, like prostaglandins (PGs), fatty acids, and their derivatives, as well as by synthetic ligands. PPAR medications have been developedand discovered to be relatively safe drugs with benefits in multiple disease states including diabetes and cardiovascular disease [67]. Fibrate drugs used to treat hyperlipidemia, and thiazolidinedione drugs used to treat type 2 diabetes are potent and relatively specific ligand activators of PPAR*α* and *γ*, respectively, and are widely used clinically [68, 69]. A number of naturally-occurring PPAR ligands have been identified, including long-chain fatty acids (C16 and greater), eicosanoids such as 8(S)-HETE (PPAR*α*) and 9-and13-HODE (PPAR*γ*), and PGs such as PGA_1,_which binds to PPAR*α*, PPAR*β*/*δ*, and 15-deoxy-delta^12,14^-prostaglandin J_2_(15dPGJ_2_), which in turn binds to PPAR*γ* [70– 72]. Both the expression of PPAR and the production of their potential ligands are altered during pregnancy and its related diseases. We postulate that pathologic diversion of fatty-acid metabolism away from the production of eicosanoid ligands in preeclampsia and gestational diabetes might be corrected using synthetic ligands.

## 2. PPARs IN TROPHOBLAST INVASION AND PLACENTAL DEVELOPMENT

In first trimester, human placental bed biopsies, PPAR-*γ* is expressed predominantly in invasive trophoblasts, whereas in the second-trimester PPAR*γ* is expressed in the columns of anchoring villi and cytotrophoblasts [73, 74]. In the third trimester, PPAR*γ* principally localizes to extravillous cytotrophoblasts (EVCT) and villous syncytiotrophoblasts [75], where it appears to regulate placental hormone production and secretion. Although the focus of this review is to summarize findings on PPAR/RXR heterodimers in human placentation, much of the direct evidence for a role of these receptors in trophoblast invasion and placental development has emerged from studies in knockout mouse models. This topic is reviewed comprehensively in Schaiff et al. [3], and is summarized briefly here and in Table 3 [76–81].

PPAR*γ*/RXR*α* heterodimers play a key regulatory role in murine placental development. PPAR*γ* deficiency was shown to interfere with terminal trophoblast differentiation and placental vascularization [78]; embryos without this gene show massive placental defects that can be rescued by restoration of the trophoblast PPAR*γ* gene via tetraploid chimeras [15]. Deletion of RXR*α* and RXR*β* also leads to embryo lethality [15, 81, 83]. Both PPAR-interacting protein (PRIP) and nuclear receptor-activating protein 250 (RAP250) encode nuclear receptor coactivators that associate with PPARs, RXRs, and other nuclear receptor proteins. Genetic disruption of PRIP or RAP250 in mouse models results in embryonic lethality at postconception days 11.5 and 13.5, respectively [79, 80]. Placentas of PRIP (−/−) and RAP250 (−/−) embryos exhibited dramatically reduced spongiotrophoblast and labyrinth layers as well as failure of blood vessel maturation in the region bordering the spongiotrophoblast [79, 80].

In addition to placentation per se, PPAR*γ* appears to play an important role in the uterine preparation for embryonic implantation. Peeters et al. demonstrated that PPAR*γ* ligands reduced the production of the endometrial angiogenic factor VEGF, and postulated that this pathway might influence early embryonic vascularization [84]. By contrast, PPAR*γ* agonists induce angiogenesis in cardiac myofibroblasts, smooth muscle cells, and macrophages [85– 87]. Recent preliminary data by our lab and others suggest that the PPAR*γ* system also stimulates VEGF expression in trophoblast (JEG-3) cells (Depoix et al., unpublished).

The functional role of PPAR*γ* activity is well studied in trophoblast physiology (Table 4). PPAR*γ* agonists inhibit invasion of cultured EVCT isolated from human first-trimester placenta, whereas PPAR*γ* antagonists promoted EVCT invasion and repressed the PPAR*γ* agonist-mediated effects [78]. PPAR*γ* controls mucin (MUC)-1 transcription and regulates maternal-fetal transport in mouse models [88]. Moreover, PPAR*γ* and RXR*α* play a role in human chorionic gonadotropin (hCG) expression, trophoblast differentiation, and regulation of fatty acid transport and storage in human placental trophoblasts [89, 90]. PPAR*γ* diminishes leptin-induced inflammatory responses in the human placenta [91] and inhibits PAPP-A expression [92].

Regulation of PPAR*γ* in human placental tissues is thought to occur through natural ligands (e.g., 15dPGJ2, 9-HODE, 13-HODE, and 15-HETE) through direct binding to the receptor’s ligand binding pocket [11, 100]. These ligands are likely to be synthesized locally within the placenta. Furthermore, crosstalk between the mitogen-activated protein kinase (MAPK) p38 and PPAR*γ* occurs within cultured trophoblast cells [101]. PPAR*γ* decreases IGFII secretion and is thought to inhibit trophoblast invasion via the PAPP-A cascade [92].

In young PPAR*α* knock out mice, no major phenotypic differences of gross pathology of internal organs were described [76, 102]. However, disturbance of the Th1/Th2 T-lymphocyte ratio, rather than placental malformation, is thought to be responsible for an increased abortion rate (20%) in PPAR*α* null mice. During normal pregnancy Th1 cytokines are downregulated and Th2 cytokines are upregulated [103].

The third distinct PPAR, PPAR*β*/*δ* also is essential for placentation as demonstrated in PPAR*β*/*δ* knockout mice (Table 3) [77], and is involved in the regulation of implantation in other animal models [17, 104, 105]. The implantation of cultured embryos is enhanced by PPAR*β*/*δ* activation and this receptor even has been postulated as a novel therapeutic target to improve clinical IVF outcomes [104]. PPAR*β*/*δ* is induced during decidualization of the implantation site and requires close contact with the blastocyst. PPAR*β*/*δ* null mice die between 9.5 to 10.5 embryonic days due to abnormal cell-cell communication at the placental-decidual interface [8].

Together these data suggest that PPARs are required not only for trophoblast invasion and differentiation but also for establishment of the placental maternal-fetal transport.

## 3. PPARs AND PREGNANCY

Based on its regulatory functions and known eicosanoid ligands, PPAR*γ* has emerged as an excellent candidate to play a role in the regulation of maternal metabolism, maintenance of uterine quiescence, and onset of labor by regulating proinflammatory cytokines and prostaglandins (Table 4). Normal pregnancy is accompanied by changes in lipid and glucose metabolism, but further dysregulation of these pathways can lead to pregnancy complications such as PE or GDM. Hence, PPAR regulators of these metabolic pathways might be expected to be important in human pregnancy.

**Figure 1:**
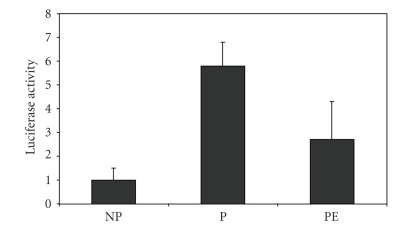
JEG-3 cells were transfected with PPRE-luciferase reporter vectors and treated with pooled sera (10%) from non-pregnant (NP), pregnant (P) and preeclamptic (PE) women. Luciferase acitivity, relative to cells treated with 10% dextran charcoal-shipped fetal calf serum (DCSS), is reported on the ordinate.

**Figure 2:**
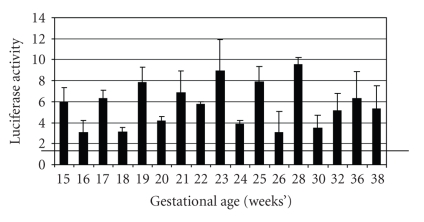
PPAR*γ* activation is present throughout the course of normal pregnancy. All serum samples were collected from the same subject and PPRE-luciferase reporter experiments were performed using 10% serum as described in Figure 1. Luciferase activity was normalized to DCSS to determine relative activation. Black horizontal bar represents the level of signaling seen with 10% serum from the same woman six weeks after delivery.

Some of our initial studies in this field were designed to screen for potential activators of PPAR*γ* in the circulation of pregnant women. Human choriocarcinoma JEG-3 cells were transfected with peroxisome-proliferator responsive reporter plasmids; and pooled sera from pregnant and nonpregnant women were added to the cell culture medium [73]. Peroxisome proliferator responsive element (PPRE) luciferase reporter activation was dramatically increased by sera from pregnant women compared to nonpregnant women (Figures 1 and 2). We showed that PPAR*γ* (and to some extent PPAR*α*) activity is increased from the earliest stages of pregnancy (Figure 2). The findings suggested that circulating PPAR*γ*-activating factors, presumably eicosanoids, were present throughout the course of gestation. We hypothesized that activation of PPAR*γ* by sera of pregnant women is a regulatory adaptation of the maternal organism to increased lipid and glucose loading in pregnancy [73].

It also has been hypothesized that PPAR*γ* activation regulates uterine quiescence by influencing Nuclear Factor-Kappa B (NF*κ*B) and cyclooxygenase (COX-2) expression [96, 97, 106]. Reciprocal expression of PPAR*γ* and (COX)-2 in human term placenta suggests a role of the PPAR system in the initiation of labor [98]. Under conditions of high PPAR*γ* expression, antiinflammatory actions dominate; however, with onset of labor PPAR*γ* levels drop and COX-2 concomitantly increases in the fetal membranes [98]. Elevated COX-2 activity in the human amnion is observed in the settings of term and idiopathic preterm labor, contributing to the generation of uterotonic prostaglandins (PGs), which are known to participate in parturition [107]. PPAR*γ* ligands have been shown to antagonize NF-*κ*B activation and reduce inflammatory cytokine gene expression (IL-1*β*, IL-6, IL-10 and TNF-*α*) and COX-2 [108]. Both natural (e.g., 15dPGJ2) and synthetic ligands (e.g., troglitazone) were shown to have anti-inflammatory effects in human gestational tissues, significantly decreasing basal and LPS-stimulated PGE_2_ and PGF_2*α*_ release from placenta and amnion [108]. PGF_2*α*_ , also a marker of oxidative stress, is increased in women with preeclampsia [109]. Given the inflammatory changes observed in pregnancy-specific diseases, a potential role of PPAR agonist treatment has been entertained for the treatment of PE, GDM, and other pregnancy-specific diseases such as the prevention of preterm labor [96].

PPAR*α* and *β*/*δ* also play a role in maintaining pregnancy and parturition. PPAR*α* and *β*/*δ* are expressed in the amnion, choriodecidua, and villous placental tissues. Data from PPAR*α* knockout mice suggest that PPAR*α* maintains pregnancy by stimulating a Th2 cytokine response [76]. In normal pregnancy, expression of PPAR*α* declines in the choriodecidua with the onset of labor [99]. By contrast, PPAR*β*/*δ* expression, which is temporally upregulated between the first and third trimester of pregnancy [99], increases further in the amnion coincidental with the onset of labor [99].

Few studies have elucidated substantial risk of PPAR agonists during pregnancy in animal models, but these drugs carry a “C” classification from the FDA. For example, rosiglitazone did not damage blastocyst development in vitro or harm mouse fetuses when given during murine pregnancy [110]. While the use of rosiglitazone during pregnancy is generally considered to be safe [110]; more data need to be acquired before these drugs can be recommended.

## 4. PPARs and PREGNANCY-SPECIFIC DISEASES

Failure of metabolic adaptation to pregnancy can result in pregnancy-specific complications such as PE and GDM. We and others have postulated that angiogenic factors and cytokines that lead to pathological gestational changes are likely to be regulated by the PPAR system (Table 5).

### 4.1. PPARs and preeclampsia

PE is a multifactorial, pregnancy-related disorder that is defined by new-onset hypertension and proteinuria after 20 weeks of gestation [117]. PE is a common cause of maternal and infant morbidity and mortality worldwide, and is responsible for about 20% of pregnancy-related maternal deaths in the US [118]. Women with PE have increased insulin resistance as well as hypertriglyceridemia relative to normal pregnant women [119]. To date, no effective treatment has been found that either prevents or reverses the development of the disease. Modern concepts of PE pathophysiology invoke a two-stage process. The first stage is believed to be initiated by impaired trophoblast invasion and abnormal uterine vessel remodeling. The second stage is postulated to result from circulating factors claimed to be derived from the ischemic placenta that stimulate an inflammatory activation of maternal vascular endothelial cells. PE presents clinically in the second or third trimester, however, fundamental inflammatory and angiogenic biomarkers in the serum are detectable as early as the first trimester in women with PE. Elevated concentrations of IL-2, TNF*α*, and sVEGFR-1 and reduced concentrations of PlGF, IGFBP-1, and HLA-G in the maternal serum precede the clinical manifestations of PE [119–123].

While the cause of PE remains unknown, several environmental and genetic risk factors have been identified (Table 2). Relevant to this review are hypertension, diabetes, and high (>29) body mass index (BMI) [47, 124,125]. Black race also appears to be a risk factor for PE [126] although this may be confounded by increased rates of the above risk factors. Key inflammatory and angiogenic pathways involved in the pathogenesis of PE are regulated by the PPAR system, which itself is influenced by environmental and genetic factors. We believe that exogenous and endogenous lipid regulators of PPAR play a role in maternal metabolism and immune functionin normal and pathological pregnancies. For example, dietary factors and physical activity that modulate the PPAR system have been shown to reduce the risk and course of PE (Table 2).

Similarly, genetic variations in the PPAR*γ* gene have been proposed to modify the risk of PE. For example, the Pro467Leu mutation of PPAR*γ* [127–129] is a dominant negative mutant resulting from a C-to-T transition in exon 6. A report of two individuals (one woman, one man) with this mutation showed that they developed type 2 diabetes at young ages (26 and 27 years at diagnosis), as well as early hypertension (37 and 27 years at diagnosis). Intriguingly, the woman had two pregnancies, both of which were complicated by severe PE. The Pro12Ala polymorphism occurs in PPAR*γ*2 [130], a second isoform of PPAR*γ* that is expressed mainly in adipose tissue. This mutation is the result of a C-to-G transversion in exon B. This is by far the most studied allelic variation in any PPAR, and occurs at a rate of about 12% in the Caucasian US population. While the resulting phenotype is highly diverse and even apparently contradictory, it appears that the penetrance of this mutation is influenced by other genetic, environmental, ethnic, and gender differences. The studies generally agree that the presence of the Ala allele is associated with increased BMI, an independent risk factor for PE. Thus, this polymorphism is a candidate affecting pregnancy outcome. Preliminary data of a study on the PPAR gene variations (in PPAR gene) showed no association with PE or severity of PE in a Finnish population [131]. Further studies on the association of PPAR *α*, *β*, and *γ* gene variations of mothers and offspring and pregnancy-specific diseases need to be performed in different ethnic populations.

**Figure 3:**
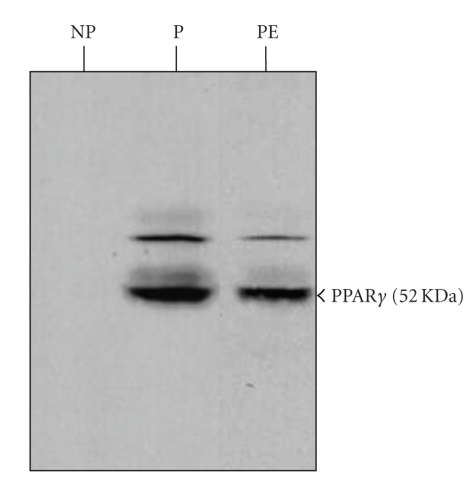
Immunoblot of JEG-3 cells treated with pooled sera (10%) from nonpregnant (NP), pregnant (P), and preeclamptic (PE) women. Cell lysates were analyzed using a specific mouse anti-human PPAR*γ* monoclonal antibody. Equal amounts of protein (50 *μ*g) were loaded into each lane. Factors in pregnant serum up-regulate JEG-3 PPAR*γ* expression. A decrease in PPAR*γ* protein was observed in cells exposed to PE sera (PE) compared to sera from normal pregnant women (P).

PE is marked by hyperlipidemia, and is characterized by a state of oxidative stress. Circulating lipids in PE women are more highly oxidized, and oxidized low-density lipoproteins (oxLDLs), in particular, are highly elevated [132]. Given the circulating plasma lipid disturbances in PE, our group performed experiments comparing sera from normal and PE patients. We found that serum from women with severe PE had reduced levels of PPAR activating lipids compared with serum of parity and gestational age-matched women and also diminished the expression of PPAR*γ* in trophoblast cells (Figures 1 and 3) [111]. The reduction of transcriptional activity observed in preeclamptic women’s sera was shown for PPAR*γ* and PPAR*α*, however not for PPAR*β*/*δ* or RXR. The reduction in potential circulating PPAR activatorswas observed weeks and sometimes months before the onset of maternal symptoms and clinicaldiagnosis of PE [133]. Our results are consistent with other clinical evidence that antiinflammatory regulation is challenged and further compromised in the maternal syndrome of PE. Normal pregnancy manifests as a physiologic inflammatory state postulated to be tolerated to serve the nutritional needs of the fetus, whereas, in PE regulatory inflammatory mechanisms are excessively amplified, leading to vascular damage in the mother [133]. In this “hyperinflammatory” state of PE [134], the cytokines TNF*α* and IL-1*β* which are typically controlled by the NF-*κ*B pathway in a negative-feedback loop with PPAR, are elevated [26, 60, 119]. Elevated inflammatory parameters in PE accompany altered levels of PG metabolites and circulating fatty acids. As noted, PG metabolites as well as fatty acids are important ligands of the PPAR system [135]. PG metabolism is altered during normal pregnancy with levels of vasorelaxants suchas prostacyclin increasing, whereas vasoconstrictive prostaglandin levelstend to be suppressed [136]. Failure of these alterations have been suggested to lead to pregnancy complications (e.g., PE) [137]. For example, PGF_2*α*_, which itself is stimulated by factorsin the plasma of women with PE [138], can inhibit PPAR*γ* effects [135]. Levels of circulating free fatty acids are in the normal range duringmost of pregnancy, but rise dramatically during the final weeks of pregnancy and drop precipitously at term [136]. In PE these levels are increased from 20 weeks’ gestation [133, 139]. We postulate that altered PG metabolism in this setting [138] results in decreased PPAR*γ* ligation and subsequent cytokine activation. If this proposal is supported by more data, the use of PPAR ligands might be proposed to ameliorate symptoms such as hypertension and inflammation. Unfortunately, at present, the mechanism and site of this salutary of PPAR ligand effect remain unknown in pregnancy, confounded by PPAR expression in many cell types, including endothelial cells.

### 4.2. PPARs and gestational diabetes

During normal pregnancy, maternal lipid, and glucose metabolism is profoundly altered [140]. The developing fetus uses glucose as its predominant energy source, which puts a continuous demand on the mother to provide this substrate [141]. This constant need for glucose results in frequent hypoglycemia and postprandial hyperglycemia during normal pregnancy [141]. Problems with energy metabolism such as GDM are not uncommon and are often observed in susceptible women at this time. GDM is defined as any degreeof glucose intolerance with onset or first recognition during pregnancy. In women with GDM, defective *β*-cells function cannot adequately compensate for free fatty acid-mediated insulin resistance [142]. As elsewhere in our society, the incidence of obesity, diabetes, and gestational diabetes mellitus are increasing in the pregnant population [143]. In the United States, the incidence of obesity among pregnant women ranges from 18.5% to 38.3% [144]; obesity comprises a major risk factor for GDM [145]. Morphological changes have been identified in the syncytiotrophoblast, cytotrophoblast, trophoblastic basement membrane, and fetal vessels within the placentae of these cases [146]. GDM is associated with several severe neonatal complications (such as macrosomia, brachial plexus palsy, premature delivery, IUGR, and intrauterine death) and maternal birth injuries also are common [125, 147]. Furthermore, GDM has emerged as a risk factor for the development of diabetes mellitus type 2 (DM2) and cardiovascular disease in later life and shares a number of epidemiologic, pathophysiologic, and genetic characteristics with DM2 [148]. GDM also has detrimental effects on the postnatal infants [149].

The PPAR system regulates the metabolic and pathways involved in the establishment of GDM. PPAR-agonists have antidiabetogenic, antiinflammatory, and antioxidant effects, which are all potentially beneficial in the treatment of GDM [5].

Environmental factors, such as diet and exercise and genetic factors influence PPAR*α*, *γ* activity [130, 150] as well as the risk for insulin resistance and GDM (Table 2). Exercise activity initiated prepregnancy was shown to reduce the risk of GDM and its complications [40, 41, 44, 151,152]. Nutritional counseling, moderate physical exercise, weight loss, and diet are successful therapies in some women with GDM, improving glycemic control, reducing the incidence of LGA infants, and decreasing the need for cesarean deliveries for cephalopelvic disproportion [41, 153].

Candidate genes for GDM risk include TNF*α*, *β*3 adrenoreceptor (ADRB3), and PPAR*α* and *γ*. The PPAR*γ* Pro12Ala polymorphism was not associated with increased insulin resistance in Turkish women with GDM, however it was associated with weight gain [112]. The PPAR*γ* coactivator-1alpha (PGC-1) polymorphism also failed to be associated with the development of GDM [154]. More studies on the association of various genetic PPAR*α* and *γ* variants and GDM in different ethnic populations will be of interest.

15dPGJ_2_ is a potent antiinflammatory agent that represses the expression of a number of inflammatory genes and regulating factors including the transcription factor NF-*κ*B [33, 108]. The concentration of 15dPGJ_2_ was reduced in placentae from diabetic rats (Table 5) [95]. Placental 15dPGJ_2_ was noted to be diminished in women with gestational and pregestational diabetes when compared to controls, whereas levels of nitric oxide (a stimulator of placental invasiveness, differentiation, and proliferation) were higher in term placental explants from diabetic patients when compared to controls [113]. As PPAR*γ* can prevent nitric oxide overproduction in placenta from pregestational diabetic women [113], it may have the potential to improve fetal outcome in this condition.

Sulfonylurea agents including gliumepiride and glibenclamide exhibit PPAR*γ* activity [155]. A randomized controlled trial to test the effectiveness and safety of the sulfonylurea agent glyburide in the management of women with GDM showed similar efficacy to insulin treatment [156]. Both the insulin- and glyburide-treated women were able to achieve satisfactory glucose control and had similar perinatal outcome [156].

### 4.3. PPARs and other pregnancy-specific diseases

Trophoblast research has emphasized the similarities between the proliferative, migratory, andinvasive properties of placental cells and those of cancer cells [157]. PPAR*γ*, PPAR*β*/*δ*, and RXR appear to be linked to gestational trophoblastic neoplasms, conditions associated with malignant trophoblast behavior [114]. PPAR*γ* agonists inhibit invasion of normal extravillous cytotrophoblast isolated from human first-trimester placenta, and PPAR activity has been shown to be downregulated in trophoblastic diseases including hydatidiform mole and choriocarcinoma [114].

PPAR*γ* has an effect on fetal and placental size influencing intrauterine growth. In an intrauterine growth restriction (IUGR) model, glucocorticoids inhibited fetal and placental growth partly by suppression of PPAR*γ* in the labyrinth zone of the placenta [158]. Activation of PPAR*γ* in the labyrinth trophoblasts is hypothesized to induce angiogenic factors and stimulate the growth of fetal blood vessels, thereby promoting placental growth. However, treatment of pregnant mice with rosiglitazone led to reduced thickness of the spongiotrophoblast layer and the surface area of labyrinthine vasculature, and it altered expression of proteins implicated in placental development [159].

In vitro and in vivo experiments as well as animal models studies suggest a link between the PPAR system and gestational duration, preterm labor, and birth weight [116]. Variations in the PPAR genes influence other pregnancy-related mechanisms including birth weight and gestational duration. In an Irish population, the PPAR*γ* Ala12 allele was associated with shorter gestational duration [116].

PPAR ligands regulate apoptotic mechanisms involved in rupture of the fetal membranes and may play a role in preterm delivery, a condition associated with increased risk of neonatal sepsis and newborn trauma [160]. 15d-PGJ_2_induced morphological characteristics of apoptosis within 2 hours in an amniotic cell line [160]. In addition, ciglitizone also induced apoptosis, whereas rosiglitazone had no effect on cell viability [160]. Prevention of apoptosis may have therapeutic potential in preterm labor and premature rupture of the membranes and necessitates further investigations.

Interestingly, PPAR*α* deficiency is associated with miscarriage, neonatal mortality, and a shift from Th2 to a Th1 cytokine phenotype [76]. Th1 predominant immunity is closely associated with inflammation, endothelial dysfunction, and pregnancy complications. For example, interferon*γ* is significantly reduced in the spleens of PPAR*α* null mice [76]. Twenty percent of PPAR*α* knockout mice aborted, and offspring of PPAR-*α* null mice exhibited increased neonatal mortality (13.3%). However the mechanism whereby PPAR*α* induces a Th2 phenotype shift remains to be determined. PPAR*γ* ligands also were shown to decrease production of inflammatory ligands in activated macrophages and T cells and to induce a shift from Th1 to Th2 cytokine phenotype [161, 162].

## 5. CONCLUSIONS

PPARs are involved in trophoblast invasion, placental development, parturition, and pregnancy-specific diseases, particularly PE and GDM. The role of the PPAR system in pregnancy under physiologic and pathologic conditions has remained partly unclear due to lack of knowledge about endogenous PPAR ligands. Pharmacological ligand research is ahead of the identification of physiologic ligands. Partially characterized inflammatory, angiogenic, and metabolic disturbances in pregnancy-related diseases suggest that these synthetic PPAR agonists may be of potential use in these conditions. Ongoing basic studies have elucidated the metabolic, antiinflammatory, and angiogenic benefits of PPAR*α*/*β*/*δ* and PPAR*γ*/*β*/*δ* dual agonists and PPAR pan agonists for treatment purposes. However, some experimental and clinical data have uncovered unfortunate side effects of PPAR ligands, including cancer progression and increased cardiac event rates. New generations of PPAR modulators are under development and these promise to be more receptor-specific, and hopefully will activate only a specific subset of target genes and metabolic pathways to reduce untoward side effects. The potential role of PPARs in regulation of inflammation and angiogenesis is intriguing and warrants further studies. We submit that PPAR agonists may become beneficial drugs for pregnancy-specific diseases, once their risks have been fully evaluated.

## Figures and Tables

**Table 1:** Effects of physiological and pathophysiological conditions on PPAR.

Influence on PPAR action			
Conditions	PPAR-action	Model	Reference
Diabetes	Increases PPAR*γ* in skeletal muscle	Murine	Park et al. [22]

Age	Increases PPAR*γ* in subcutaneous fat in older man	Human	Imbeault et al. [23]
	Decreases PPAR*α* in heart	Murine	Iemitsu et al. 2002 [24]

Hypertension	Increases PPAR*α* and *γ* in aorta and mesenteric arteries	Murine	Diep and Schiffrin [25]

Diet	Soy extract increases PPAR*α* and *γ* in macrophages	In vitro	Mezei et al. [28]
	High-fat diet increases adipose tissue expression of PPAR*γ* and induces PPAR*γ*2 mRNA expression in liver (obese mice)	Murine	Vidal-Puig et al. [26]
	Hyperlipid diet reduces PPAR*γ* in colonic epithelium	Murine	Delage et al. [29]
	Low-calorie diet decreases PPAR*γ* in subcutaneous fat	Human	Bastard et al. [27]

Exercise	Increases PPAR*γ* DNA binding activity in fat depots	Murine	Petridou et al. [30]
	Increases PPAR*α* in heart	Murine	Iemitsu et al. [24]
	Increases PPAR*β*/*δ* in skeletal muscle	Human	Fritz et al. [34]

Obesity	Increases of PPAR*γ*2 and PPAR*γ*2/PPAR*γ*1 ratio in adipose tissue	Human	Vidal-Puig et al. [31]

Metabolic syndrome	Dominant-negative mutation in PPAR*γ* induces metabolic syndrome	Human	Savage et al. [35]

Insulin resistance (IR)	Pioglitazone ameliorates IR	Murine	Ding et al. [33]
	PPAR*γ* Ala allele protects against hyperinsulinemia	Human	Jaziri et al. [32]

Vitamin A	Increases PPAR*γ* in colonic mucosa	Murine	Delage et al. [29]

**Table 2:** Effects of metabolic conditions on pregnancy-specific diseases (GDM: gestational diabetes mellitus; PE: preeclampsia; IUGR: Intrauterine growth restriction; −: reduced risk; +: increased risk).

Influence on pregnancy-specific diseases				
Conditions	GDM	PE	IUGR	Reference
Diabetes	—	+	—	Ostlund et al. [36]
Advanced maternal age	+	+	+	Delbaere et al. [53] Odibo et al. [37]
Hypertension	—	+	+	Sibai et al. [38]
Optimal nutrition	−	−	−	Artal et al. [41] Saftlas et al. [43] Scholl et al. [39]

Optimal exercise	−	−	—	Artal et al. [41] Zhang et al. [44] Sorensen et al. [42] Saftlas et al. [43]

Obesity	+	+	+	Cedergren [48] Saftlas et al. [43] O’Brien et al. [47] Ros et al. [45] Sebire et al. [46] Bodnar et al. [49]

Metabolic syndrome	+	+	+	Ray et al. [50]
Insulin resistance	—	+	—	Wolf et al. [51]
Periconceptional multivitamins	—	−	—	Bodnar et al. [52]

**Table 3:** PPAR knock out models and placental pathology (PRIP: peroxisome proliferator-activated receptor-(PPAR) interacting protein; RAP 250: nuclear receptor-activating protein 250).

PPAR knockout model	Placental pathology	Lethality	Reference
PPAR*α*	No significant effect on placentation	20%	Yessoufou et al. [76]
PPAR*β*/*δ*	Poor placentation	>90%	Barak et al. [77]
PPAR*γ*	Poorly developed labyrinth	100%	Barak et al. [15] Kubota et al. [82]
PPAR*γ* coactivator PRIP	Reduced spongiotrophoblast layer	100%	Zhu et al. [79]
PPAR*γ* coactivator RAP250	Reduced spongiotrophoblast layer	100%	Antonson et al. [80]
RXR*α* or *β*	Lack of labyrinth zone	100%	Sapin et al. [81]

**Table 4:** PPAR action in trophoblast development and placental function (MUC-1: mucin-1; EVCT: extravillous cytotrophoblast; hCG: human chorionic gonadotropin; Th2 T-helper 2 cell).

PPAR action in trophoblast development and placentation			
PPAR	PPAR action	Model	Reference
PPAR*γ*	Inhibits EVCT invasion	In vitro	Fournier et al. [78]
	Promotes trophoblast differentiation hCG secretion	In vitro	Tarrade et al. [89]
	Induces hCG production	In vitro	Schild et al. [93]
	Antiinflammatory	In vitro	Lappas et al. [91]
	Regulates fatty acid transport	In vitro	Schaiff et al. [90]
	Increases VEGF expression	In vitro	Depoix, unpublished
	Terminal differentiation, placental vascularization	Murine	Barak et al. [15]
	Controls MUC-1 expression	Murine	Shalom-Barak et al. [88]
	Stimulates trophoblast maturation	Murine	Asami-Miyagishi et al. [94]
	Modulates placental lipid metabolism	Murine	Capobianco et al. [95]

PPAR*β*/*δ*	Promotes placental development	Murine	Nadra et al. [8]

PPAR*α*	Regulates placental lipid transfer	Murine/Human	Wang et al. [74]

PPAR action in pregnancy			

PPAR*γ*	Antiinflammatory	In vitro	Lappas et al. [96]
	Involved in inflammatory control and remodeling in the placenta	In vitro	Marvin et al. [97]
	Increased circulating PPAR*γ* activators in normal pregnancy	In vitro/human	Waite et al. [73]
	Decreases in fetal membrane with labor	Human	Dunn-Albanese et al. [98]

PPAR*β*/*δ*	Increases in amnion with labor	Human	Berry et al. [99]

PPAR*α*	Stimulates Th2 cytokine pattern during pregnancy	Murine	Yessoufou et al. [76]
	Declines in choriodecidua with labor	Human	Berry et al. [99]

**Table 5:** PPAR in pregnancy-specific diseases.

PPAR	PPAR-action	Disease	Model	Reference
PPAR*γ*	Reduced circulating PPAR*γ* activators in serum from women with PE	PE	In vitro	Waite et al. [111]
	Placental 15dPGJ_2_level are decreased in diabetes	GDM	Murine	Capobianco et al. [95]
	Association of PPAR-*γ*2 Pro12Ala with weight gain	GDM	Human	Tok et al. [112]
	Placental 15dPGJ_2_levels are decreased	GDM	Human	Javerbaum et al. [113]
	Decreased	Hydatidiform mole	Human	Capparuccia et al. [114]
	Decreased	Choriocarcinoma	Human	Capparuccia et al. [114]
	Placental PPAR expression is not involved	IUGR	Human	Rodie et al. [115]
	Association of PPAR-*γ*2 Pro12Ala polymorphism	Preterm birth	Human	Meirhaeghe et al. [116]

PPAR*α*	Lack of PPAR-*α* upregulates Th1 cytokines	Abortion/neonatal mortality	Murine	Yessoufou et al. [76]
